# Supplementary material for: Selective Flamingo Medium for the Isolation of Aspergillus fumigatus
Source: Microorganisms. 2021 May 27;9(6):1155. doi: 10.3390/microorganisms9061155 (PMC8228204; doi:10.3390/microorganisms9061155)
Supplement: Supplementary file 1 [file microorganisms-09-01155-s001.zip › microorganisms-1217730-supplementary.pdf]

microorganisms

**Table S1.** Total counts of *A. fumigatus* (CFU/g) detected in various samples and surface area covered by Mucorales (%) using six different isolation media for each sample. Three technical replicates were used for each sample on each medium.

| Type of sample | Sample ID | Replicates | CFU/g | Medium | Surface area covered by Mucorales |
|---|---|---|---|---|---|
| plant waste | S127 | R1 | 7.00E+06 | MEA+C+S | 70.00% |
| plant waste | S127 | R2 | 7.00E+06 | MEA+C+S | 60.00% |
| plant waste | S127 | R3 | 5.60E+06 | MEA+C+S | 74.00% |
| plant waste | S127 | R1 | 4.00E+06 | SCG+C | 87.00% |
| plant waste | S127 | R2 | 3.60E+06 | SCG+C | 70.00% |
| plant waste | S127 | R3 | 5.40E+06 | SCG+C | 68.00% |
| plant waste | S127 | R1 | 9.00E+06 | DG18 | 10.00% |
| plant waste | S127 | R2 | 9.60E+06 | DG18 | 20.00% |
| plant waste | S127 | R3 | 8.00E+06 | DG18 | 15.00% |
| plant waste | S127 | R1 | 1.78E+07 | Flamingo | 0.00% |
| plant waste | S127 | R2 | 1.40E+07 | Flamingo | 0.00% |
| plant waste | S127 | R3 | 1.60E+07 | Flamingo | 0.00% |
| plant waste | S127 | R1 | 1.58E+07 | M-RB | 0.00% |
| plant waste | S127 | R2 | 1.60E+07 | M-RB | 0.00% |
| plant waste | S127 | R3 | 1.70E+07 | M-RB | 0.00% |
| plant waste | S127 | R1 | 1.60E+07 | MEA-RB | 10.00% |
| plant waste | S127 | R2 | 1.72E+07 | MEA-RB | 15.00% |
| plant waste | S127 | R3 | 1.46E+07 | MEA-RB | 20.00% |
| plant waste | S128 | R1 | 2.40E+07 | MEA+C+S | 60.00% |
| plant waste | S128 | R2 | 2.26E+07 | MEA+C+S | 76.00% |
| plant waste | S128 | R3 | 2.00E+07 | MEA+C+S | 58.00% |
| plant waste | S128 | R1 | 2.46E+07 | SCG+C | 20.00% |
| plant waste | S128 | R2 | 2.56E+07 | SCG+C | 30.00% |
| plant waste | S128 | R3 | 2.60E+07 | SCG+C | 43.00% |
| plant waste | S128 | R1 | 2.20E+06 | DG18 | 60.00% |
| plant waste | S128 | R2 | 5.80E+06 | DG18 | 48.00% |
| plant waste | S128 | R3 | 4.00E+06 | DG18 | 50.00% |

| | | | | | |
|---|---|---|---|---|---|
| plant waste | S128 | R1 | 2.56E+07 | Flamingo | 5.00% |
| plant waste | S128 | R2 | 2.70E+07 | Flamingo | 4.00% |
| plant waste | S128 | R3 | 2.82E+07 | Flamingo | 3.00% |
| plant waste | S128 | R1 | 2.70E+07 | M-RB | 3.00% |
| plant waste | S128 | R2 | 2.54E+07 | M-RB | 4.00% |
| plant waste | S128 | R3 | 2.72E+07 | M-RB | 5.00% |
| plant waste | S128 | R1 | 1.96E+07 | MEA-RB | 45.00% |
| plant waste | S128 | R2 | 2.00E+07 | MEA-RB | 53.00% |
| plant waste | S128 | R3 | 2.24E+07 | MEA-RB | 44.00% |
| plant waste | S131 | R1 | 1.96E+07 | MEA+C+S | 80.00% |
| plant waste | S131 | R2 | 1.52E+07 | MEA+C+S | 70.00% |
| plant waste | S131 | R3 | 1.70E+07 | MEA+C+S | 87.00% |
| plant waste | S131 | R1 | 2.00E+07 | SCG+C | 70.00% |
| plant waste | S131 | R2 | 1.74E+07 | SCG+C | 74.00% |
| plant waste | S131 | R3 | 1.84E+07 | SCG+C | 67.00% |
| plant waste | S131 | R1 | 2.00E+05 | DG18 | 80.00% |
| plant waste | S131 | R2 | 4.00E+05 | DG18 | 70.00% |
| plant waste | S131 | R3 | 0.00E+00 | DG18 | 80.00% |
| plant waste | S131 | R1 | 2.24E+07 | Flamingo | 5.00% |
| plant waste | S131 | R2 | 2.20E+07 | Flamingo | 4.00% |
| plant waste | S131 | R3 | 2.00E+07 | Flamingo | 3.00% |
| plant waste | S131 | R1 | 2.26E+07 | M-RB | 5.00% |
| plant waste | S131 | R2 | 2.02E+07 | M-RB | 3.00% |
| plant waste | S131 | R3 | 2.16E+07 | M-RB | 2.00% |
| plant waste | S131 | R1 | 1.82E+07 | MEA-RB | 80.00% |
| plant waste | S131 | R2 | 1.74E+07 | MEA-RB | 78.00% |
| plant waste | S131 | R3 | 1.78E+07 | MEA-RB | 70.00% |
| wood | S150 | R1 | 2.88E+03 | MEA+C+S | 10.00% |
| wood | S150 | R2 | 3.24E+03 | MEA+C+S | 5.00% |
| wood | S150 | R3 | 3.36E+03 | MEA+C+S | 10.00% |
| wood | S150 | R1 | 4.56E+03 | SCG+C | 10.00% |
| wood | S150 | R2 | 3.24E+03 | SCG+C | 10.00% |
| wood | S150 | R3 | 3.72E+03 | SCG+C | 20.00% |

| | | | | | |
|---|---|---|---|---|---|
| wood | S150 | R1 | 4.80E+02 | DG18 | 20.00% |
| wood | S150 | R2 | 1.08E+03 | DG18 | 20.00% |
| wood | S150 | R3 | 9.60E+02 | DG18 | 31.00% |
| wood | S150 | R1 | 6.00E+03 | Flamingo | 0.00% |
| wood | S150 | R2 | 3.72E+03 | Flamingo | 0.00% |
| wood | S150 | R3 | 5.52E+03 | Flamingo | 0.00% |
| wood | S150 | R1 | 5.16E+03 | M-RB | 0.00% |
| wood | S150 | R2 | 4.68E+03 | M-RB | 0.00% |
| wood | S150 | R3 | 4.56E+03 | M-RB | 0.00% |
| wood | S150 | R1 | 3.36E+03 | MEA-RB | 10.00% |
| wood | S150 | R2 | 3.72E+03 | MEA-RB | 10.00% |
| wood | S150 | R3 | 4.20E+03 | MEA-RB | 0.00% |
| wood | S158 | R1 | 4.80E+03 | MEA+C+S | 80.00% |
| wood | S158 | R2 | 2.28E+03 | MEA+C+S | 90.00% |
| wood | S158 | R3 | 4.20E+03 | MEA+C+S | 76.00% |
| wood | S158 | R1 | 3.72E+03 | SCG+C | 90.00% |
| wood | S158 | R2 | 3.24E+03 | SCG+C | 80.00% |
| wood | S158 | R3 | 2.16E+03 | SCG+C | 80.00% |
| wood | S158 | R1 | 1.20E+03 | DG18 | 60.00% |
| wood | S158 | R2 | 1.68E+03 | DG18 | 10.00% |
| wood | S158 | R3 | 1.92E+03 | DG18 | 40.00% |
| wood | S158 | R1 | 3.24E+03 | Flamingo | 5.00% |
| wood | S158 | R2 | 3.84E+03 | Flamingo | 3.00% |
| wood | S158 | R3 | 4.44E+03 | Flamingo | 2.00% |
| wood | S158 | R1 | 4.20E+03 | M-RB | 3.00% |
| wood | S158 | R2 | 3.00E+03 | M-RB | 5.00% |
| wood | S158 | R3 | 4.44E+03 | M-RB | 1.00% |
| wood | S158 | R1 | 4.20E+03 | MEA-RB | 10.00% |
| wood | S158 | R2 | 3.60E+03 | MEA-RB | 13.00% |
| wood | S158 | R3 | 2.52E+03 | MEA-RB | 15.00% |
| wood | S157 | R1 | 9.36E+03 | MEA+C+S | 68.00% |
| wood | S157 | R2 | 8.04E+03 | MEA+C+S | 80.00% |
| wood | S157 | R3 | 9.84E+03 | MEA+C+S | 70.00% |

| | | | | | |
|---|---|---|---|---|---|
| wood | S157 | R1 | 7.08E+03 | SCG+C | 78.00% |
| wood | S157 | R2 | 8.28E+03 | SCG+C | 80.00% |
| wood | S157 | R3 | 7.32E+03 | SCG+C | 59.00% |
| wood | S157 | R1 | 6.48E+03 | DG18 | 50.00% |
| wood | S157 | R2 | 4.32E+03 | DG18 | 60.00% |
| wood | S157 | R3 | 5.76E+03 | DG18 | 70.00% |
| wood | S157 | R1 | 1.07E+04 | Flamingo | 0.00% |
| wood | S157 | R2 | 8.52E+03 | Flamingo | 0.00% |
| wood | S157 | R3 | 9.48E+03 | Flamingo | 0.00% |
| wood | S157 | R1 | 1.02E+04 | M-RB | 0.00% |
| wood | S157 | R2 | 1.01E+04 | M-RB | 0.00% |
| wood | S157 | R3 | 8.64E+03 | M-RB | 0.00% |
| wood | S157 | R1 | 5.76E+03 | MEA-RB | 59.00% |
| wood | S157 | R2 | 6.48E+03 | MEA-RB | 65.00% |
| wood | S157 | R3 | 6.12E+03 | MEA-RB | 69.00% |
| soil | S137 | R1 | 8.04E+03 | MEA+C+S | 80.00% |
| soil | S137 | R2 | 9.36E+03 | MEA+C+S | 70.00% |
| soil | S137 | R3 | 7.08E+03 | MEA+C+S | 67.00% |
| soil | S137 | R1 | 9.60E+03 | SCG+C | 70.00% |
| soil | S137 | R2 | 8.04E+03 | SCG+C | 75.00% |
| soil | S137 | R3 | 6.72E+03 | SCG+C | 69.00% |
| soil | S137 | R1 | 4.32E+03 | DG18 | 70.00% |
| soil | S137 | R2 | 5.16E+03 | DG18 | 80.00% |
| soil | S137 | R3 | 5.76E+03 | DG18 | 75.00% |
| soil | S137 | R1 | 1.20E+04 | Flamingo | 0.00% |
| soil | S137 | R2 | 1.16E+04 | Flamingo | 0.00% |
| soil | S137 | R3 | 1.04E+04 | Flamingo | 0.00% |
| soil | S137 | R1 | 1.18E+04 | M-RB | 0.00% |
| soil | S137 | R2 | 1.03E+04 | M-RB | 0.00% |
| soil | S137 | R3 | 1.31E+04 | M-RB | 0.00% |
| soil | S137 | R1 | 8.04E+03 | MEA-RB | 50.00% |
| soil | S137 | R2 | 8.88E+03 | MEA-RB | 58.00% |
| soil | S137 | R3 | 8.28E+03 | MEA-RB | 45.00% |

| | | | | | |
|---|---|---|---|---|---|
| soil | S138 | R1 | 1.08E+04 | MEA+C+S | 90.00% |
| soil | S138 | R2 | 9.60E+03 | MEA+C+S | 87.00% |
| soil | S138 | R3 | 9.48E+03 | MEA+C+S | 80.00% |
| soil | S138 | R1 | 1.19E+04 | SCG+C | 69.00% |
| soil | S138 | R2 | 1.07E+04 | SCG+C | 76.00% |
| soil | S138 | R3 | 1.24E+04 | SCG+C | 80.00% |
| soil | S138 | R1 | 1.08E+04 | DG18 | 70.00% |
| soil | S138 | R2 | 9.48E+03 | DG18 | 80.00% |
| soil | S138 | R3 | 8.28E+03 | DG18 | 80.00% |
| soil | S138 | R1 | 1.44E+04 | Flamingo | 0.00% |
| soil | S138 | R2 | 1.56E+04 | Flamingo | 0.00% |
| soil | S138 | R3 | 1.67E+04 | Flamingo | 0.00% |
| soil | S138 | R1 | 1.63E+04 | M-RB | 0.00% |
| soil | S138 | R2 | 1.66E+04 | M-RB | 0.00% |
| soil | S138 | R3 | 1.55E+04 | M-RB | 0.00% |
| soil | S138 | R1 | 1.20E+04 | MEA-RB | 50.00% |
| soil | S138 | R2 | 1.18E+04 | MEA-RB | 45.00% |
| soil | S138 | R3 | 9.36E+03 | MEA-RB | 65.00% |
| soil | S151 | R1 | 9.60E+03 | MEA+C+S | 40.00% |
| soil | S151 | R2 | 9.12E+03 | MEA+C+S | 56.00% |
| soil | S151 | R3 | 8.40E+03 | MEA+C+S | 39.00% |
| soil | S151 | R1 | 8.04E+03 | SCG+C | 30.00% |
| soil | S151 | R2 | 8.88E+03 | SCG+C | 28.00% |
| soil | S151 | R3 | 6.96E+03 | SCG+C | 39.00% |
| soil | S151 | R1 | 2.16E+03 | DG18 | 20.00% |
| soil | S151 | R2 | 3.00E+03 | DG18 | 30.00% |
| soil | S151 | R3 | 3.48E+03 | DG18 | 40.00% |
| soil | S151 | R1 | 9.36E+03 | Flamingo | 5.00% |
| soil | S151 | R2 | 1.07E+04 | Flamingo | 3.00% |
| soil | S151 | R3 | 1.12E+04 | Flamingo | 2.00% |
| soil | S151 | R1 | 1.04E+04 | M-RB | 2.00% |
| soil | S151 | R2 | 8.88E+03 | M-RB | 1.00% |
| soil | S151 | R3 | 1.08E+04 | M-RB | 1.00% |

| | | | | | |
|---|---|---|---|---|---|
| soil | S151 | R1 | 8.04E+03 | MEA-RB | 20.00% |
| soil | S151 | R2 | 8.88E+03 | MEA-RB | 27.00% |
| soil | S151 | R3 | 8.28E+03 | MEA-RB | 32.00% |
| grass/root | S139 | R1 | 6.00E+02 | MEA+C+S | 80.00% |
| grass/root | S139 | R2 | 7.20E+02 | MEA+C+S | 70.00% |
| grass/root | S139 | R3 | 6.00E+02 | MEA+C+S | 80.00% |
| grass/root | S139 | R1 | 4.80E+02 | SCG+C | 50.00% |
| grass/root | S139 | R2 | 1.20E+02 | SCG+C | 30.00% |
| grass/root | S139 | R3 | 2.40E+02 | SCG+C | 60.00% |
| grass/root | S139 | R1 | 2.40E+02 | DG18 | 20.00% |
| grass/root | S139 | R2 | 2.40E+02 | DG18 | 10.00% |
| grass/root | S139 | R3 | 4.80E+02 | DG18 | 0.00% |
| grass/root | S139 | R1 | 7.20E+02 | Flamingo | 0.00% |
| grass/root | S139 | R2 | 3.60E+02 | Flamingo | 0.00% |
| grass/root | S139 | R3 | 6.00E+02 | Flamingo | 0.00% |
| grass/root | S139 | R1 | 9.60E+02 | M-RB | 0.00% |
| grass/root | S139 | R2 | 3.60E+02 | M-RB | 0.00% |
| grass/root | S139 | R3 | 6.00E+02 | M-RB | 0.00% |
| grass/root | S139 | R1 | 6.00E+02 | MEA-RB | 10.00% |
| grass/root | S139 | R2 | 3.60E+02 | MEA-RB | 20.00% |
| grass/root | S139 | R3 | 7.20E+02 | MEA-RB | 0.00% |
| grass/root | S140 | R1 | 9.60E+03 | MEA+C+S | 35.00% |
| grass/root | S140 | R2 | 1.08E+04 | MEA+C+S | 30.00% |
| grass/root | S140 | R3 | 9.48E+03 | MEA+C+S | 29.00% |
| grass/root | S140 | R1 | 9.36E+03 | SCG+C | 50.00% |
| grass/root | S140 | R2 | 7.80E+03 | SCG+C | 60.00% |
| grass/root | S140 | R3 | 7.20E+03 | SCG+C | 56.00% |
| grass/root | S140 | R1 | 2.88E+03 | DG18 | 30.00% |
| grass/root | S140 | R2 | 4.20E+03 | DG18 | 50.00% |
| grass/root | S140 | R3 | 4.56E+03 | DG18 | 40.00% |
| grass/root | S140 | R1 | 1.20E+04 | Flamingo | 0.00% |
| grass/root | S140 | R2 | 1.18E+04 | Flamingo | 0.00% |
| grass/root | S140 | R3 | 9.72E+03 | Flamingo | 0.00% |

| | | | | | |
|---|---|---|---|---|---|
| grass/root | S140 | R1 | 1.30E+04 | M-RB | 0.00% |
| grass/root | S140 | R2 | 1.25E+04 | M-RB | 0.00% |
| grass/root | S140 | R3 | 1.20E+04 | M-RB | 0.00% |
| grass/root | S140 | R1 | 1.18E+04 | MEA-RB | 0.00% |
| grass/root | S140 | R2 | 1.09E+04 | MEA-RB | 0.00% |
| grass/root | S140 | R3 | 1.02E+04 | MEA-RB | 0.00% |
| grass/root | S143 | R1 | 1.20E+02 | MEA+C+S | 10.00% |
| grass/root | S143 | R2 | 1.20E+02 | MEA+C+S | 40.00% |
| grass/root | S143 | R3 | 1.20E+02 | MEA+C+S | 50.00% |
| grass/root | S143 | R1 | 1.20E+02 | SCG+C | 30.00% |
| grass/root | S143 | R2 | 0.00E+00 | SCG+C | 0.00% |
| grass/root | S143 | R3 | 1.20E+02 | SCG+C | 20.00% |
| grass/root | S143 | R1 | 1.20E+02 | DG18 | 20.00% |
| grass/root | S143 | R2 | 0.00E+00 | DG18 | 30.00% |
| grass/root | S143 | R3 | 2.40E+02 | DG18 | 10.00% |
| grass/root | S143 | R1 | 2.40E+02 | Flamingo | 2.00% |
| grass/root | S143 | R2 | 1.20E+02 | Flamingo | 1.00% |
| grass/root | S143 | R3 | 1.20E+02 | Flamingo | 3.00% |
| grass/root | S143 | R1 | 1.20E+02 | M-RB | 0.00% |
| grass/root | S143 | R2 | 1.20E+02 | M-RB | 0.00% |
| grass/root | S143 | R3 | 2.40E+02 | M-RB | 0.00% |
| grass/root | S143 | R1 | 0.00E+00 | MEA-RB | 20.00% |
| grass/root | S143 | R2 | 2.40E+02 | MEA-RB | 30.00% |
| grass/root | S143 | R3 | 1.20E+02 | MEA-RB | 20.00% |
| ditch water | S159 | R1 | 1.56E+04 | MEA+C+S | 20.00% |
| ditch water | S159 | R2 | 1.45E+04 | MEA+C+S | 26.00% |
| ditch water | S159 | R3 | 1.40E+04 | MEA+C+S | 30.00% |
| ditch water | S159 | R1 | 1.20E+04 | SCG+C | 15.00% |
| ditch water | S159 | R2 | 1.34E+04 | SCG+C | 17.00% |
| ditch water | S159 | R3 | 1.39E+04 | SCG+C | 20.00% |
| ditch water | S159 | R1 | 5.04E+03 | DG18 | 20.00% |
| ditch water | S159 | R2 | 3.96E+03 | DG18 | 30.00% |
| ditch water | S159 | R3 | 4.68E+03 | DG18 | 15.00% |

| | | | | | |
|---|---|---|---|---|---|
| ditch water | S159 | R1 | 1.44E+04 | Flamingo | 3.00% |
| ditch water | S159 | R2 | 1.51E+04 | Flamingo | 4.00% |
| ditch water | S159 | R3 | 1.38E+04 | Flamingo | 5.00% |
| ditch water | S159 | R1 | 1.42E+04 | M-RB | 5.00% |
| ditch water | S159 | R2 | 1.43E+04 | M-RB | 4.00% |
| ditch water | S159 | R3 | 1.51E+04 | M-RB | 5.00% |
| ditch water | S159 | R1 | 1.20E+04 | MEA-RB | 16.00% |
| ditch water | S159 | R2 | 1.32E+04 | MEA-RB | 15.00% |
| ditch water | S159 | R3 | 1.18E+04 | MEA-RB | 10.00% |
| ditch water | S160 | R1 | 8.40E+03 | MEA+C+S | 90.00% |
| ditch water | S160 | R2 | 1.08E+04 | MEA+C+S | 79.00% |
| ditch water | S160 | R3 | 9.12E+03 | MEA+C+S | 87.00% |
| ditch water | S160 | R1 | 9.24E+03 | SCG+C | 89.00% |
| ditch water | S160 | R2 | 7.80E+03 | SCG+C | 93.00% |
| ditch water | S160 | R3 | 9.12E+03 | SCG+C | 92.00% |
| ditch water | S160 | R1 | 4.20E+03 | DG18 | 60.00% |
| ditch water | S160 | R2 | 6.00E+03 | DG18 | 40.00% |
| ditch water | S160 | R3 | 5.40E+03 | DG18 | 35.00% |
| ditch water | S160 | R1 | 1.44E+04 | Flamingo | 0.00% |
| ditch water | S160 | R2 | 1.56E+04 | Flamingo | 0.00% |
| ditch water | S160 | R3 | 1.42E+04 | Flamingo | 0.00% |
| ditch water | S160 | R1 | 1.42E+04 | M-RB | 0.00% |
| ditch water | S160 | R2 | 1.52E+04 | M-RB | 0.00% |
| ditch water | S160 | R3 | 1.38E+04 | M-RB | 0.00% |
| ditch water | S160 | R1 | 1.32E+04 | MEA-RB | 10.00% |
| ditch water | S160 | R2 | 1.18E+04 | MEA-RB | 20.00% |
| ditch water | S160 | R3 | 1.07E+04 | MEA-RB | 14.00% |
| ditch water | S161 | R1 | 3.60E+02 | MEA+C+S | 20.00% |
| ditch water | S161 | R2 | 6.00E+02 | MEA+C+S | 10.00% |
| ditch water | S161 | R3 | 3.60E+02 | MEA+C+S | 15.00% |
| ditch water | S161 | R1 | 3.60E+02 | SCG+C | 10.00% |
| ditch water | S161 | R2 | 3.60E+02 | SCG+C | 23.00% |
| ditch water | S161 | R3 | 3.60E+02 | SCG+C | 18.00% |

| | | | | | |
|---|---|---|---|---|---|
| ditch water | S161 | R1 | 1.20E+02 | DG18 | 10.00% |
| ditch water | S161 | R2 | 2.40E+02 | DG18 | 20.00% |
| ditch water | S161 | R3 | 0.00E+00 | DG18 | 30.00% |
| ditch water | S161 | R1 | 3.60E+02 | Flamingo | 5.00% |
| ditch water | S161 | R2 | 3.60E+02 | Flamingo | 1.00% |
| ditch water | S161 | R3 | 3.60E+02 | Flamingo | 1.00% |
| ditch water | S161 | R1 | 2.40E+02 | M-RB | 1.00% |
| ditch water | S161 | R2 | 4.80E+02 | M-RB | 2.00% |
| ditch water | S161 | R3 | 3.60E+02 | M-RB | 2.00% |
| ditch water | S161 | R1 | 3.60E+02 | MEA-RB | 7.00% |
| ditch water | S161 | R2 | 2.40E+02 | MEA-RB | 8.00% |
| ditch water | S161 | R3 | 6.00E+02 | MEA-RB | 10.00% |
| air | BT | R1 | 7.20E+03 | MEA+C+S | 5.00% |
| air | BT | R2 | 8.28E+03 | MEA+C+S | 3.00% |
| air | BT | R3 | 8.52E+03 | MEA+C+S | 7.00% |
| air | BT | R1 | 8.52E+03 | SCG+C | 8.00% |
| air | BT | R2 | 8.16E+03 | SCG+C | 6.00% |
| air | BT | R3 | 7.20E+03 | SCG+C | 5.00% |
| air | BT | R1 | 6.00E+03 | DG18 | 10.00% |
| air | BT | R2 | 5.28E+03 | DG18 | 14.00% |
| air | BT | R3 | 4.80E+03 | DG18 | 8.00% |
| air | BT | R1 | 8.16E+03 | Flamingo | 0.00% |
| air | BT | R2 | 7.80E+03 | Flamingo | 0.00% |
| air | BT | R3 | 9.00E+03 | Flamingo | 0.00% |
| air | BT | R1 | 6.96E+03 | M-RB | 0.00% |
| air | BT | R2 | 7.68E+03 | M-RB | 0.00% |
| air | BT | R3 | 8.40E+03 | M-RB | 0.00% |
| air | BT | R1 | 6.84E+03 | MEA-RB | 0.00% |
| air | BT | R2 | 7.92E+03 | MEA-RB | 0.00% |
| air | BT | R3 | 8.88E+03 | MEA-RB | 0.00% |
| air | WM | R1 | 1.61E+04 | MEA+C+S | 16.00% |
| air | WM | R2 | 1.44E+04 | MEA+C+S | 23.00% |
| air | WM | R3 | 1.32E+04 | MEA+C+S | 9.00% |

| | | | | | |
|---|---|---|---|---|---|
| air | WM | R1 | 1.61E+04 | SCG+C | 6.00% |
| air | WM | R2 | 1.56E+04 | SCG+C | 13.00% |
| air | WM | R3 | 1.45E+04 | SCG+C | 11.00% |
| air | WM | R1 | 9.60E+03 | DG18 | 20.00% |
| air | WM | R2 | 1.08E+04 | DG18 | 10.00% |
| air | WM | R3 | 9.12E+03 | DG18 | 15.00% |
| air | WM | R1 | 1.82E+04 | Flamingo | 0.00% |
| air | WM | R2 | 1.58E+04 | Flamingo | 0.00% |
| air | WM | R3 | 1.44E+04 | Flamingo | 0.00% |
| air | WM | R1 | 1.43E+04 | M-RB | 0.00% |
| air | WM | R2 | 1.20E+04 | M-RB | 0.00% |
| air | WM | R3 | 1.50E+04 | M-RB | 0.00% |
| air | WM | R1 | 1.44E+04 | MEA-RB | 0.00% |
| air | WM | R2 | 1.40E+04 | MEA-RB | 2.00% |
| air | WM | R3 | 1.55E+04 | MEA-RB | 0.00% |
| air | PT | R1 | 2.11E+04 | MEA+C+S | 8.00% |
| air | PT | R2 | 1.87E+04 | MEA+C+S | 5.00% |
| air | PT | R3 | 1.78E+04 | MEA+C+S | 3.00% |
| air | PT | R1 | 1.82E+04 | SCG+C | 10.00% |
| air | PT | R2 | 1.96E+04 | SCG+C | 19.00% |
| air | PT | R3 | 1.55E+04 | SCG+C | 12.00% |
| air | PT | R1 | 1.44E+04 | DG18 | 22.00% |
| air | PT | R2 | 1.56E+04 | DG18 | 15.00% |
| air | PT | R3 | 1.20E+04 | DG18 | 6.00% |
| air | PT | R1 | 1.78E+04 | Flamingo | 0.00% |
| air | PT | R2 | 1.84E+04 | Flamingo | 0.00% |
| air | PT | R3 | 1.68E+04 | Flamingo | 0.00% |
| air | PT | R1 | 2.08E+04 | M-RB | 0.00% |
| air | PT | R2 | 1.98E+04 | M-RB | 0.00% |
| air | PT | R3 | 1.70E+04 | M-RB | 0.00% |
| air | PT | R1 | 1.82E+04 | MEA-RB | 0.00% |
| air | PT | R2 | 1.96E+04 | MEA-RB | 3.00% |
| air | PT | R3 | 2.16E+04 | MEA-RB | 0.00% |

**Supplementary data on the statistics**

Significant difference among media in detection of *A. fumigatus* and surface area covered by Mucorales species were performed with Kruskal-Wallis test.

**Hypothesis Test Summary**

| | Null Hypothesis | Test | Sig. | Decision |
|---|---|---|---|---|
| 1 | The medians of CFU/g are the sameacross categories of Medium. | Independent-SamplesMedian Test | .001 | Reject the nullhypoth-esis. |
| 2 | The distribution of CFU/g is the sameacross catego-ries of Medium. | Independent-SamplesKruskal-Wallis Test | .004 | Reject the nullhypoth-esis. |
| 3 | The medians of Background of Mucorales species are the same acrosscategories of Medium. | Independent-SamplesMedian Test | .000 | Reject the nullhypoth-esis. |
| 4 | The distribution of Background of Mucorales species is the same acrosscategories of Medium. | Independent-SamplesKruskal-Wallis Test | .000 | Reject the nullhypoth-esis. |

Asymptotic significances are displayed. The significance level is .05.

| Sample 1-Sample 2 | Adj.Sig. |
|---|---|
| M-RB vs Flamingo | 1.000 |
| M-RB vs MEA-RB | 1.000 |
| M-RB vs DG18 | 0.002 |
| M-RB vs SCG+C | 0.314 |
| M-RB vs MEA+C+S | 0.314 |
| Flamingo vs MEA-RB | 1.000 |
| Flamingo vs DG18 | 0.000 |
| Flamingo vs SCG+C | 0.314 |
| Flamingo vs MEA+C+S | 0.814 |
| MEA-RB vs DG18 | 0.004 |
| MEA-RB vs MEA+C+S | 1.000 |
| MEA-RB vs SCG+C | 1.000 |
| DG18 vs SCG+C | 0.002 |
| DG18 vs MEA+C+S | 0.002 |
| SCG+C vs MEA+C+S | 1.000 |

| Sample 1-Sample 2 | Adj.Sig. |
|---|---|
| M-RB vs Flamingo | 1.000 |
| M-RB vs MEA-RB | 1.000 |
| M-RB vs DG18 | 0.006 |
| M-RB vs SCG+C | 1.000 |
| M-RB vs MEA+C+S | 1.000 |
| Flamingo vs MEA-RB | 1.000 |
| Flamingo vs DG18 | 0.005 |
| Flamingo vs SCG+C | 0.000 |
| Flamingo vs MEA+C+S | 0.000 |
| MEA-RB vs DG18 | 0.159 |
| MEA-RB vs MEA+C+S | 1.000 |
| MEA-RB vs SCG+C | 1.000 |
| DG18 vs SCG+C | 0.369 |
| DG18 vs MEA+C+S | 0.128 |
| SCG+C vs MEA+C+S | 1.000 |

| Sample 1-Sample 2 | Adj.Sig. |
|---|---|
| M-RB vs Flamingo | 1.000 |
| M-RB vs MEA-RB | 0.000 |
| M-RB vs DG18 | 0.000 |
| M-RB vs SCG+C | 0.000 |
| M-RB vs MEA+C+S | 0.000 |
| Flamingo vs MEA-RB | 0.000 |
| Flamingo vs DG18 | 0.000 |
| Flamingo vs SCG+C | 0.000 |
| Flamingo vs MEA+C+S | 0.000 |
| MEA-RB vs DG18 | 0.302 |
| MEA-RB vs MEA+C+S | 0.008 |
| MEA-RB vs SCG+C | 0.031 |
| DG18 vs SCG+C | 1.000 |
| DG18 vs MEA+C+S | 0.814 |
| SCG+C vs MEA+C+S | 1.000 |

| Sample 1-Sample 2 | Adj.Sig. |
|---|---|
| M-RB vs Flamingo | 1.000 |
| M-RB vs MEA-RB | 0.000 |
| M-RB vs DG18 | 0.000 |
| M-RB vs SCG+C | 0.000 |
| M-RB vs MEA+C+S | 0.000 |
| Flamingo vs MEA-RB | 0.000 |
| Flamingo vs DG18 | 0.000 |
| Flamingo vs SCG+C | 0.000 |
| Flamingo vs MEA+C+S | 0.000 |
| MEA-RB vs DG18 | 0.081 |
| MEA-RB vs MEA+C+S | 0.001 |
| MEA-RB vs SCG+C | 0.006 |
| DG18 vs SCG+C | 1.000 |
| DG18 vs MEA+C+S | 1.000 |
| SCG+C vs MEA+C+S | 1.000 |
